# Development and validation of the college students’ time management competence scale

**DOI:** 10.3389/fpsyg.2026.1885044

**Published:** 2026-09-03

**Authors:** Fang Liu, Man Li

**Affiliations:** 1 School of Education, Central China Normal University, Wuhan, Hubei, China; 2 School of Life Science and Health Engineering, Luoyang Institute of Science and Technology, Luoyang, Henan, China; 3 School of Humanities, Luoyang Institute of Science and Technology, Luoyang, Henan, China

**Keywords:** college students, scale construction, self-regulated learning, structural equation modeling, temporal organization

## Abstract

**Introduction:**

In an era characterized by rapid knowledge progression and intensified academic and social competition, time management skills have become a crucial competency for college students to manage academic responsibilities, balance various life commitments, and enhance personal competitiveness. Although current tools, such as the Adolescent Time Management Disposition Scale (ATMD), have contributed to prior studies, their dimensional frameworks may inadequately capture the psychological and behavioral characteristics of contemporary college students in digitally mediated environments. Thus, the present study aimed to develop and validate the College Students’ Time Management Competence Scale (CSTMC).

**Methods:**

A total of 861 responses were retained across two independent samples. A 30-item candidate pool was generated through a structured review of Chinese- and English-language literature on time management and self-regulated learning (SRL). A single-round structured expert review involving four experts (two linguists and two subject-matter specialists) resulted in the removal of eight items before pilot testing. The pilot form therefore contained 22 scored items and one additional unscored instructed-response attention-check item used only for response-quality screening. Of the 250 pilot questionnaires submitted, 209 responses met the response-quality criteria and constituted the exploratory factor analysis (EFA) sample; an independent sample of 652 responses constituted the confirmatory factor analysis (CFA) sample. Internal consistency, composite reliability, convergent validity, and factor-level discriminant validity were evaluated.

**Results:**

EFA supported a five-factor structure comprising (1) Sense of Time Value, (2) Time Planning, (3) Time Monitoring, (4) Time Regulation, and (5) Sense of Time Efficacy. Together, the five factors accounted for 68.847% of the total variance. The subsequent CFA showed satisfactory model fit: *χ*^2^/df = 1.8676, RMSEA = 0.037, CFI = 0.976, IFI = 0.976, and NFI = 0.949. The final 22-item scale demonstrated satisfactory psychometric properties. Cronbach’s alpha was 0.95 for the total scale and ranged from 0.810 to 0.879 across the subscales. All composite reliability (CR) values exceeded 0.70, and all average variance extracted (AVE) values exceeded 0.50, supporting internal consistency and convergent validity.

**Discussion:**

The findings provide initial evidence that the CSTMC can assess multidimensional time-management competence in college students, including time-value beliefs, planning, monitoring, regulation, and efficacy. The scale offers a theory-informed framework for identifying distinct competence profiles and for developing targeted, low-stakes educational supports, while additional criterion-related and cross-context validation remains necessary.

## 1 Introduction

Time management has evolved beyond mere scheduling and is now examined across several disciplines. In psychology, it is commonly conceptualized in terms of goal setting, planning, prioritization, and effective task execution, and it has been linked to wellbeing and quality of life (Lakein, 1973; Li, 2022). From an educational-economics perspective, time is a scarce resource whose allocation can influence the efficiency of educational investment and outcomes (Hanushek, 2006). Educational psychology further clarifies the cognitive constraints on learning through phenomena such as the spacing effect (Ebbinghaus, 1964; Cepeda et al., 2006) and cognitive load (Sweller, 1988).

In the digital era, this capability is especially important. Castells' (2000) concept of “timeless time” describes the erosion of conventional temporal and spatial boundaries in networked life (Xie, 2025), while the acceleration of social life may contribute to persistent time-related anxiety (Rosa, 2013; Gu, 2023). For college students, time is not only a medium for learning but also a resource through which identity and long-term developmental goals are organized (Zimmerman and Moylan, 2009).

The time-management competence of Chinese college students is also shaped by a distinctive educational time culture. During primary and secondary education, students often experience tightly structured schedules and substantial externally directed time investment (Sun et al., 2025). Schools organize learning through fixed timetables and examination cycles, which can support knowledge acquisition but may also limit opportunities to develop autonomous temporal decision making (Li and Sun, 2025). Prolonged reliance on externally imposed schedules may therefore leave some students underprepared for the greater temporal autonomy of university life.

Upon entering college, students encounter a substantially different temporal environment. Relaxed institutional constraints provide greater autonomy, while pervasive digital media expose students to a continuous stream of information and distractions (Wang, 2022). Without sufficiently developed internal regulatory mechanisms, students may alternate between compensatory leisure and unproductive busyness, experiencing procrastination-related anxiety while investing effort in inefficient activity patterns (Jin et al., 2024; Su and Yuan, 2023). This transition highlights the need to move beyond externally scheduling students toward helping them regulate their own time.

Existing time-management measures can be grouped into three broad traditions. Cognitive approaches assess how individuals organize and deploy time-related strategies (Britton and Tesser, 1991); behavioral approaches quantify specific time-management practices (Macan et al., 1990); and dispositional approaches assess relatively stable attitudes and tendencies. A prominent example of the third tradition is the Adolescent Time Management Disposition Scale (ATMD; Huang and Zhang, 2001), which comprises Sense of Time Value, Time Monitoring, and Sense of Time Efficacy and has been widely used in subsequent research (Zhou, 2026).

Despite its contribution, the ATMD places goal setting, planning, prioritization, time allocation, process monitoring, and feedback within an aggregated Time Monitoring dimension. This broad domain is useful for describing general disposition but offers limited diagnostic granularity for explaining the intention-action gap. A student may value planning and report making plans but still fail to enact or revise them when interruptions occur; an aggregated score cannot identify whether the difficulty lies in prospective planning, awareness of actual time use, or feedback-based adjustment.

In this study, time-management competence is therefore defined as the capacity to organize temporal resources and to monitor and regulate cognition, behavior, and emotion in pursuit of time-related goals. It is treated as a dynamic, cyclical process rather than a static trait. A contemporary college-student measure should distinguish intention from enacted competence, separate planning from execution and adjustment, and remain sensitive to the increased autonomy, fragmented schedules, and digital interruptions that characterize university life.

Time management is an essential component of self-regulated learning (SRL) and is associated with educational outcomes (Liu et al., 2026; Wolters et al., 2025). The present study therefore aimed to develop and validate the College Students’ Time Management Competence Scale (CSTMC), using SRL as its core theoretical foundation. Insights from executive-function research, management, and organizational behavior were used as supplementary background to clarify the trainability and applied relevance of time-management competence rather than as direct biological evidence for the proposed dimensions. The study examined whether the theory-derived distinctions among time value, planning, monitoring, regulation, and efficacy were supported by an interpretable empirical factor structure and adequate initial psychometric evidence.

## 2 Review of literature

### 2.1 Theoretical development and practical applications of SRL

From a psychological perspective, time management is an important component of SRL. SRL refers to learners’ active regulation of cognition, motivation, and behavior in the pursuit of learning goals (Zimmerman, 1989). Its development marked a shift from models centered primarily on external behavioral control toward accounts that recognize learners as agents in their own learning. Bandura’s social learning theory introduced an agentic account of learning that emphasized observational and self-regulatory processes (Bandura, 1977). This account was subsequently developed into social cognitive theory, which highlighted self-observation, self-evaluation, self-reaction, and reciprocal interactions among personal, behavioral, and environmental influences (Bandura, 1986). These processes established the basis for later educational models of SRL.

Building on Bandura’s work, Zimmerman applied self-regulation theory to learning and described SRL as learners’ active participation in their learning through metacognitive, motivational, and behavioral processes (Zimmerman, 1989). He identified six broad dimensions—motivation, methods, time, behavior, environment, and social influences (Zimmerman, 1998)—and developed a three-phase cyclical model comprising forethought, performance, and self-reflection (Zimmerman, 2000). Time management is one way in which this cycle is expressed in the temporal organization of learning. Related models have extended the SRL framework by emphasizing goal orientation (Pintrich, 2000), the integration of cognition and motivation (Boekaerts, 1996), and information-processing and feedback mechanisms (Winne, 2001).

Across educational settings, SRL research consistently emphasizes that effective learning depends on the coordinated use of planning, monitoring, strategy adjustment, motivation, and contextual resources. Although models differ in terminology and emphasis, they converge on the view that learners actively compare current progress with intended goals and modify their actions when discrepancies arise (Pintrich, 2000; Winne, 2001; Zimmerman, 2000).

### 2.2 Temporal organization in SRL

Within the SRL framework, time management is commonly treated as a resource-management strategy. Effective time management includes setting time frames, decomposing tasks, prioritizing activities, and monitoring progress (Zimmerman, 2002). These practices help embed cognitive and metacognitive activity within organized periods of study (Zimmerman and Risemberg, 1997). Executive-function research provides a complementary account of the cognitive processes that may support these practices, including working memory, inhibitory control, planning, and cognitive flexibility (Diamond, 2013).

Executive-function research provides a background account of why time-management competence may be trainable, rather than direct biological validation of the CSTMC dimensions. Working memory can support the maintenance of goals, priorities, and schedules; inhibitory control can support resistance to competing impulses and digital distractions; and cognitive flexibility can support replanning when circumstances change (Diamond, 2013). Experience-dependent plasticity further suggests that these functions can improve through repeated practice (Kolb and Gibb, 2011). However, the present study did not collect neurocognitive-task or neural data. Accordingly, the neuroscientific literature is used only to establish theoretical plausibility for the malleability of planning and regulation, not to claim a one-to-one neural mapping or empirical confirmation of the scale’s factor structure.

### 2.3 Enhancing the methodology for time management interventions

The effects of time-management interventions are complex. Early research emphasized practical strategies such as calendar use, scheduling, and task decomposition, which may support short-term task completion and perceived control over time (Macan, 1994). However, the effects of tool-focused training may be difficult to sustain when such training is not supported by motivation and broader self-regulatory processes. Wolters et al. (2025) situate time management within the SRL framework and describe it as an adaptive process through which students organize time and effort, manage distractions, evaluate their use of time, and modify strategies in pursuit of academic goals. Instruction in prospective planning, performance monitoring, and post-task reflection may therefore enhance the adaptability of students’ time management (Zimmerman, 2002). Such instruction may draw on executive functions related to goal maintenance, inhibitory control, and flexible adjustment; however, the present study did not collect neurocognitive or neural data and therefore provides no direct evidence of changes in specific brain systems.

### 2.4 Assessment of time management proficiency and the concerns addressed in this research

The traditional three-dimensional model underlying the ATMD provides a stable trait-based account of time-management disposition. However, when this model is treated as a direct measure of time-management competence and used to guide specific interventions, its conceptual granularity is limited. A competence-oriented measure should connect relatively stable beliefs with observable strategic and regulatory functions. The present study therefore re-examines the broad ATMD Time Monitoring dimension through the three interrelated phases of SRL, providing a more fine-grained framework for identifying where difficulties arise in the time-management process.

The three-dimensional ATMD model comprises Sense of Time Value, Time Monitoring, and Sense of Time Efficacy. Its Time Monitoring dimension includes goal setting, planning, prioritization, time allocation, outcome monitoring, and feedback, thereby combining anticipatory planning, observation during performance, and strategy modification. Executive-function research suggests that planning, monitoring, and adaptive control are theoretically distinguishable functions (Shallice and Burgess, 1991; Koechlin et al., 2003). These functions also fail in different ways: a student may formulate an unrealistic schedule, fail to notice a deviation, or notice the deviation but fail to adjust. The ATMD can describe broad tendencies, but it cannot readily locate which of these process-specific difficulties is most prominent.

Conceptually, planning addresses what should be done and when, whereas control addresses whether implementation remains aligned with the plan and how deviations should be corrected. Distinguishing these functions enables the framework to correspond more closely with the SRL cycle of forethought, performance, and reflection and provides more precise targets for intervention.

To avoid conflating observation with action, the present study defines Time Monitoring and Time Regulation according to the direction and function of metacognitive information flow. Nelson and Narens (1990) conceptualized metacognition as an interaction between an object level, at which ongoing cognition and behavior occur, and a meta-level, at which those activities are represented, evaluated, and regulated. Monitoring conveys information from the object level to the meta-level, whereas control conveys regulatory commands from the meta-level back to the object level. Applied to time management, Time Monitoring refers to acquiring and appraising information about actual time use and task progress, such as recording where time was spent, recognizing deviations from a schedule, and judging whether progress is on pace. It therefore answers the diagnostic question, “What is happening, and how does it compare with the intended state?”

Time Regulation, by contrast, refers to the context-specific regulation of behavior and strategy required to initiate, maintain, restore, or revise action in accordance with temporal goals and time-management plans. It includes beginning planned actions without unnecessary delay, maintaining plan-consistent behavior, managing time-relevant distractions, and making feedback-based adjustments such as reprioritizing tasks, rescheduling activities, changing work pace, or reallocating time. It therefore answers not only the control question, “What should I change now?” but also the implementation question, “How should I regulate my behavior so that action remains aligned with the temporal goal?”

**Figure 1:**
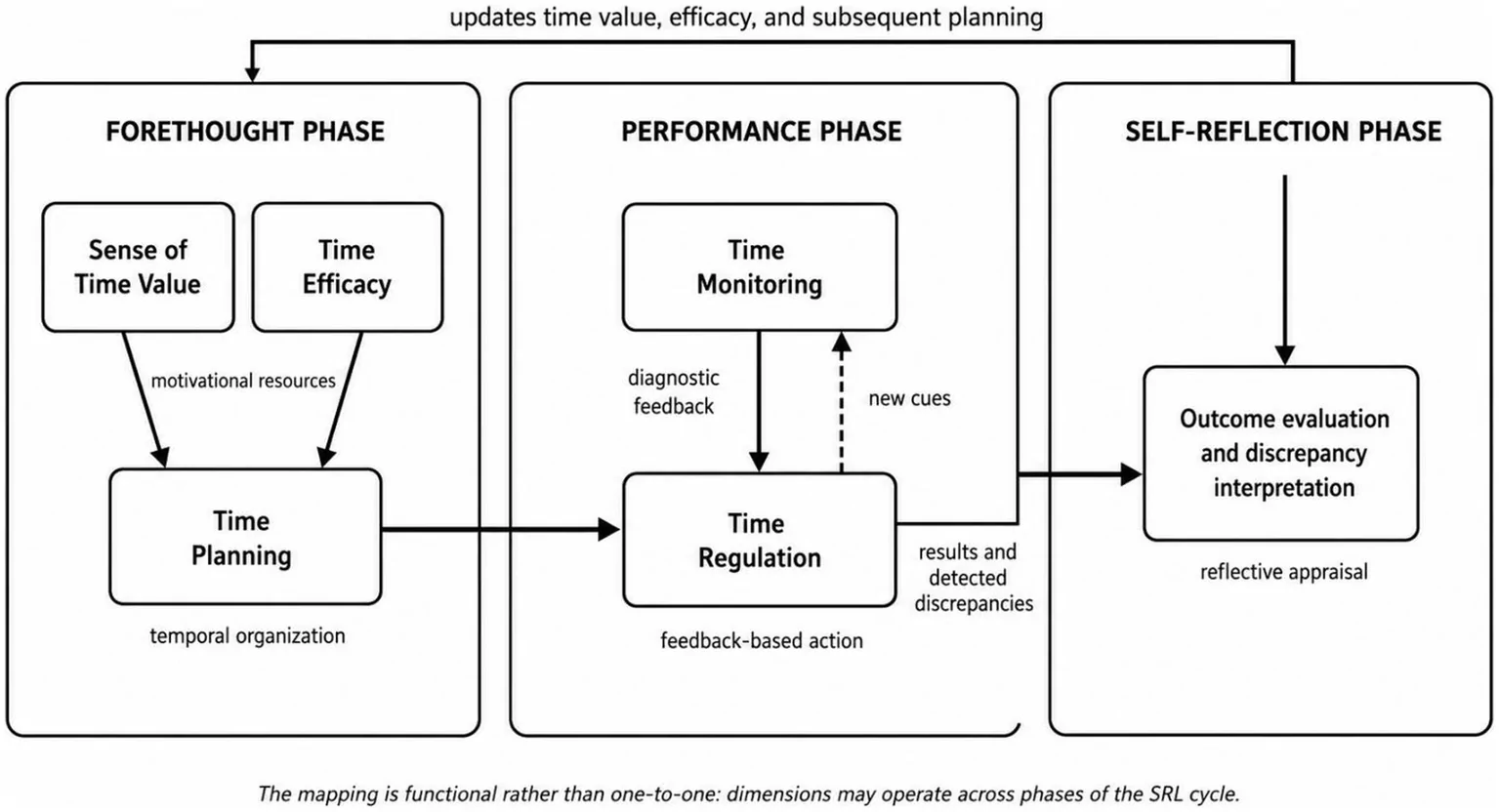
Theory-driven correspondence between the SRL cycle and the five CSTMC dimensions. Sense of Time Value and Time Efficacy support goal engagement; Time Planning organizes temporal resources; Time Monitoring generates diagnostic feedback during performance; Time Regulation implements feedback-based adjustments; and reflective appraisal updates subsequent value, efficacy, and planning. The correspondence is functional rather than a strict one-to-one mapping. Source: Generated by the author.

The two processes are reciprocally linked and may occur in rapid succession, but their functional outputs differ: monitoring generates diagnostic feedback, whereas regulation initiates, maintains, restores, or changes the course of action. Empirical metacognition research likewise indicates that monitoring can guide control and that control can generate cues for subsequent monitoring (Koriat et al., 2006). Accordingly, Time Planning captures anticipatory goal setting and temporal organization; Time Monitoring captures ongoing observation, recording, and discrepancy detection; and Time Regulation captures implementation and feedback-based adaptation during execution. This decomposition preserves the cyclical character of SRL while providing separate intervention targets for failures of awareness and failures of behavioral adjustment. This theory-driven correspondence is summarized in Figure 1.

## 3 Materials and methods

### 3.1 Research group

The survey encompassed six provinces, municipalities, and autonomous regions in eastern, central, and southwestern China, excluding Tibet, on the basis of the study’s sampling plan and data availability. Two independent samples were used to develop and validate the factor structure. At the pilot stage, a stratified sampling strategy was used to recruit 250 college students, who submitted pilot questionnaires. The questionnaire contained 22 scored CSTMC items and one instructed-response attention-check item. The attention-check item was excluded from scoring, item analysis, exploratory factor analysis (EFA), confirmatory factor analysis (CFA), and reliability estimation.

Before the psychometric analyses, 41 pilot responses were excluded according to prespecified response-quality criteria: 19 respondents failed the instructed-response attention-check item, 11 completed the questionnaire in less than one third of the median completion time, and 11 displayed long-string responding or near-zero response variability across the substantive items. The remaining 209 responses were retained for item analysis and EFA, yielding an 83.6% usable-response rate. The independent CFA sample comprised 652 responses. Thus, 250 refers to the number of submitted pilot questionnaires, whereas 209 and 652 are the analysis sample sizes for EFA and CFA, respectively. The demographic characteristics of both samples are presented in Table 1.

**Table 1:** Demographic characteristics of participants.

Items	Categories	EFA sample (*N* = 209)		CFA sample (*N* = 652)	
		*n*	%	*n*	%
Gender	Male	117	55.87	351	53.83
	Female	92	44.13	301	46.17
Grade	Freshman	52	24.85	168	25.77
	Sophomore	52	24.88	165	25.31
	Junior	52	24.88	162	24.85
	Senior+	53	25.36	157	24.08
Institution type	Undergraduate	101	48.32	320	49.08
	Vocational	108	51.68	332	50.92

In the EFA sample, 55.98% of participants were male and 44.02% were female (Table 1). Students classified as seniors or above comprised 25.36% of the sample. Vocational-college students represented 51.67%, and students from undergraduate institutions represented 48.33%.

In the CFA sample, 53.83% of participants were male and 46.17% were female (Table 1). First-year students comprised 25.77% of the sample. Vocational-college students represented 50.92%, and students from traditional undergraduate institutions represented 49.08%.

### 3.2 Scale development and item generation

The scale-development procedure followed the eight-step guidance of DeVellis and Thorpe (2021). For clarity, the procedure is reported below in three chronological stages: literature-based item generation, a single-round structured expert review, and pilot psychometric evaluation followed by independent-sample CFA.

#### 3.2.1 Literature review and initial item pool

To define the construct domain, the research team conducted a structured, theory-oriented literature review rather than a systematic review or meta-analysis. Searches were conducted in the Web of Science Core Collection and the China National Knowledge Infrastructure (CNKI) from database inception to 31 March 2026, followed by a supplementary update on 15 May 2026. The English search terms were: (“time management” OR “time management disposition” OR “time management competence” OR “temporal organization”) AND (“self-regulated learning” OR metacognit* OR planning OR monitoring OR regulation OR “executive function”) AND (“college student*” OR “university student*” OR undergraduate*). Corresponding Chinese search terms used in CNKI included “时间管理” (time management), “时间管理倾向” (time management disposition), “时间管理能力” (time management competence), “自我调节学习” (self-regulated learning), “元认知” (metacognition), “时间规划” (time planning), “时间监控” (time monitoring), “时间调节” (time regulation), “大学生” (college students), and “高校学生” (university students). Search terms were combined according to the syntax of each database. Search terms were combined and adapted according to the syntax and search functions of each database. The reference lists of eligible publications and established instruments, including the Adolescent Time Management Disposition Scale (ATMD), were also searched manually. Supplementary targeted searches were conducted using Google Scholar to locate relevant books, book chapters, and sources not consistently indexed in the selected databases. Publisher webpages and DOI records were used only to retrieve full texts and verify bibliographic information rather than as additional systematic search databases.

Publications or instruments were included when they (a) conceptualized or measured time-management-related cognition, motivation, behavior, or self-regulation; (b) reported identifiable dimensions, behavioral indicators, or item content; (c) were applicable or adaptable to college students or learning contexts; and (d) provided sufficient information to support construct definition or item generation. Sources were excluded when they (a) focused exclusively on organizational scheduling or employee productivity without content transferable to student learning; (b) assessed only objective time allocation rather than individual competence, disposition, or self-regulation; (c) duplicated an earlier version of the same instrument; (d) lacked accessible construct definitions or item-level information; or (e) were non-academic commentaries without an identifiable theoretical or empirical basis. Candidate statements were mapped onto the provisional theoretical domains. Semantically redundant, double-barrelled, overly general, or contextually unsuitable statements were merged, rewritten, or excluded, producing an initial pool of 30 items.

#### 3.2.2 Single-round structured expert review and content refinement

The initial 30-item pool underwent a single-round structured expert review. Because there was no iterative feedback-and-rerating process, the procedure is described as a structured expert review rather than a Delphi study. Four experts were purposively invited: two linguists with experience in questionnaire wording and two subject-matter specialists with research experience in educational psychology, SRL, time management, and scale development. Each expert provided an overall three-point judgment for every item: 1 = inappropriate and recommended for deletion, 2 = appropriate after revision, and 3 = appropriate for retention. In making this judgment, the experts considered construct relevance, correspondence with the proposed dimension, wording clarity, and appropriateness for college students. They also provided open-ended comments on semantic redundancy, double-barrelled wording, and potential overlap between dimensions.

Item decisions were made by the research team after jointly considering the experts’ ratings and written comments, with priority given to adequate construct coverage and clear dimensional correspondence. Numerical ratings were not used as an automatic criterion for item deletion. Items were retained when their content was relevant, clearly worded, and assignable to a single proposed dimension. Items were revised when the underlying content was relevant but the wording was ambiguous or insufficiently adapted to college students. Items were deleted when they were semantically redundant, weakly aligned with the intended construct, double-barrelled, overly scenario-specific, contaminated by adjacent constructs, or not unambiguously assignable to one dimension.

Particular attention was given to operationally distinguishing Time Monitoring from Time Regulation during item construction and expert review. Monitoring items were restricted to observation, recording, awareness, appraisal, or discrepancy detection without requiring a corrective response. Regulation items, by contrast, required an explicit change in strategy or behavior triggered by disruption or feedback. Thus, an item such as “I can clearly know where my time has gone” was classified as monitoring, whereas “When my plan is disrupted, I can adjust it in a timely manner” was classified as regulation. Items that simultaneously described awareness and corrective action were treated as potentially cross-dimensional and were rewritten to represent a single process or removed when a clear single-process formulation could not be achieved. This wording rule was used to minimize semantic overlap between the two subscales.

Through this theory-informed qualitative review, eight items were deleted, reducing the candidate pool from 30 to 22 scored items before pilot testing. The procedure was structured but did not constitute a formal Delphi consensus process, and no quantitative content-validity index was calculated. Detailed item-level review decisions and the corresponding rationales for item deletion are presented in Supplementary Table S1.

#### 3.2.3 Pilot form and psychometric evaluation

The expert-approved pilot form contained 23 administered statements: 22 scored items and one unscored instructed-response attention-check item used only for response-quality screening. The 22 scored items used a five-point Likert response format ranging from Strongly Disagree (1) to Strongly Agree (5). The attention-check item was excluded from all psychometric analyses. The scored structure comprised Sense of Time Value (3 items), Time Planning (4 items), Time Monitoring (4 items), Time Regulation (6 items), and Sense of Time Efficacy (5 items), for a total of 22 scored items.

A total of 250 students completed and submitted the pilot questionnaire. After response-quality screening, 41 cases were excluded: 19 failed the instructed-response attention-check item, 11 had completion times shorter than one third of the median completion time, and 11 exhibited long-string responding or near-invariant response patterns. The remaining 209 responses were retained for analysis. Before EFA, item discrimination, item-total correlations, changes in Cronbach’s alpha after item deletion, and item communalities were examined. Items with primary factor loadings below 0.40, cross-loadings above 0.32, or theoretically uninterpretable factor membership were flagged for substantive review rather than automatic deletion (Tabachnick and Fidell, 2019). No scored item was deleted during pilot item analysis or EFA, and all 22 scored items were retained for CFA in the independent validation sample. At the CFA stage, item performance was evaluated with reference to standardized factor loadings, residuals, overall model fit, theoretical relevance, and content coverage rather than any single statistical cutoff. Detailed EFA and CFA results are reported in Sections 4.2 and 4.3.

## 4 Results

### 4.1 Item analysis of the 22 scored items

The pilot questionnaire contained 23 administered statements: 22 scored CSTMC items and one unscored attention-check item. The attention-check item was used only for response-quality screening and was excluded from item and factor analyses. Item analysis evaluated the discrimination and internal coherence of the 22 scored items using the upper-lower group method, item-total correlations, changes in Cronbach’s alpha after item deletion, and common-factor indices (DeVellis and Thorpe, 2021). Independent-samples *t* tests comparing the upper and lower 27% groups showed significant differences for all 22 scored items (*p* < 0.01), with *t* values greater than 3. Item-total correlations ranged from 0.678 to 0.827, and no coefficient was below 0.40. Removing any item did not improve the overall Cronbach’s alpha. Common-factor indices ranged from 0.597 to 0.762. Taken together, these results indicated adequate item discrimination and internal consistency, and no scored item met the prespecified criteria for deletion before EFA.

### 4.2 EFA

All 22 scored items were entered into EFA; the unscored attention-check item was excluded. Principal component extraction with Varimax rotation was used to investigate the dimensional structure. Five components had eigenvalues greater than 1. The first component had an eigenvalue of 7.695 and explained 34.980% of the variance; the second had an eigenvalue of 2.415 and explained 10.979%; the third had an eigenvalue of 1.902 and explained 8.645%; the fourth had an eigenvalue of 1.600 and explained 7.274%; and the fifth had an eigenvalue of 1.533 and explained 6.969%. Together, the five components explained 68.847% of the total variance. The Kaiser–Meyer–Olkin (KMO) measure of sampling adequacy was 0.883, indicating that the sample was suitable for factor analysis.

**Figure 2:**
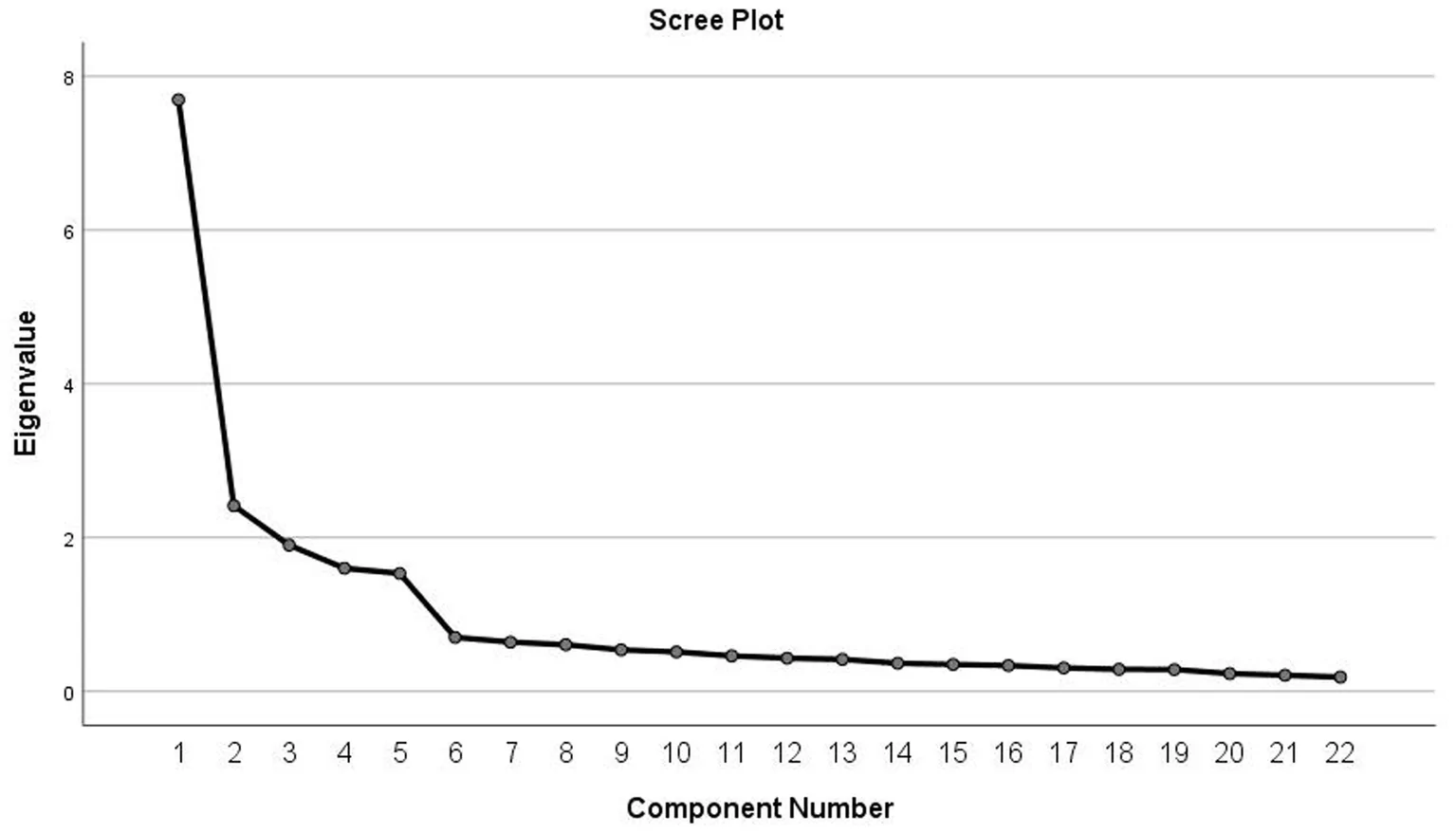
Scree plot output. Source: Generated by the author.

The eigenvalues, percentages of variance explained, and cumulative variance are summarized in Table 2, while the scree plot is presented in Figure 2. The eigenvalue pattern and scree plot jointly supported the retention of five components. These five factors explained 68.847% of the total variance, indicating that the retained factor solution accounted for a substantial proportion of variance in the observed items. Factor retention was further supported by the interpretability of the factor structure and its consistency with the proposed theoretical framework (Fabrigar et al., 1999).

**Table 2:** Results of exploratory factor analysis (EFA) for the CSTMC (*N* = 209).

Total variance explained									
Component	Initial eigenvalues			Extraction sums of squared loadings			Rotation sums of squared loadings		
	Total	% of Variance	Cumulative %	Total	% of Variance	Cumulative %	Total	% of Variance	Cumulative %
1	7.695	34.980	34.980	7.695	34.980	34.980	3.860	17.543	17.543
2	2.415	10.979	45.958	2.415	10.979	45.958	3.283	14.921	32.464
3	1.902	8.645	54.603	1.902	8.645	54.603	2.956	13.435	45.899
4	1.600	7.274	61.877	1.600	7.274	61.877	2.652	12.055	57.954
5	1.533	6.969	68.847	1.533	6.969	68.847	2.396	10.893	68.847
6	0.702	3.189	72.035						
7	0.640	2.908	74.943						
8	0.606	2.757	77.700						
9	0.539	2.449	80.149						
10	0.512	2.327	82.476						
11	0.461	2.094	84.570						
12	0.432	1.962	86.531						
13	0.416	1.889	88.420						
14	0.365	1.661	90.081						
15	0.349	1.588	91.669						
16	0.335	1.525	93.194						
17	0.304	1.382	94.576						
18	0.286	1.302	95.878						
19	0.283	1.286	97.164						
20	0.230	1.047	98.211						
21	0.208	0.948	99.159						
22	0.185	0.841	100.000						

The rotated solution supported a five-factor structure for the 22 scored items. Time Regulation comprised six items (Items 5–10), with primary loadings from 0.664 to 0.790 and communalities from 0.578 to 0.731. Sense of Time Efficacy comprised five scored items (Items 11–15), with primary loadings from 0.678 to 0.822 and communalities from 0.554 to 0.771. Time Monitoring comprised four items (Items 19–22), with primary loadings from 0.740 to 0.815 and communalities from 0.729 to 0.764. Time Planning comprised four items (Items 1–4), with primary loadings from 0.695 to 0.810 and communalities from 0.622 to 0.717. Sense of Time Value comprised three items (Items 16–18), with primary loadings from 0.779 to 0.839 and communalities from 0.732 to 0.776. No item was deleted because of cross-loading or any other EFA criterion; the same 22 scored items that entered EFA were retained in the five-factor solution. The separate attention-check item was not included in this numbering or in the factor analysis. The complete rotated loading matrix is presented in Table 3.

**Table 3:** Rotated component matrix.

Items	Component					Extraction
	1	2	3	4	5	
Q1	0.117	0.015	0.169	**0.810**	0.134	0.717
Q2	0.031	0.180	0.107	**0.760**	0.245	0.683
Q3	0.198	0.301	0.086	**0.695**	−0.054	0.622
Q4	0.130	0.081	−0.023	**0.807**	0.007	0.676
Q5	**0.664**	0.234	0.269	0.057	0.192	0.607
Q6	**0.790**	0.192	0.199	0.146	0.090	0.731
Q7	**0.751**	0.138	0.078	0.173	0.197	0.657
Q8	**0.728**	0.108	0.263	0.054	0.241	0.672
Q9	**0.737**	0.017	0.163	0.005	0.084	0.578
Q10	**0.777**	0.140	0.149	0.210	0.077	0.696
Q11	0.148	**0.732**	0.164	0.210	0.145	0.650
Q12	0.190	**0.678**	0.116	0.208	0.021	0.554
Q13	0.129	**0.819**	0.128	0.153	0.209	0.771
Q14	0.070	**0.822**	0.141	0.078	0.158	0.732
Q15	0.115	**0.739**	0.028	−0.028	−0.140	0.581
Q16	0.266	0.085	0.155	0.071	**0.818**	0.776
Q17	0.219	0.183	0.159	0.137	**0.779**	0.732
Q18	0.145	0.010	0.122	0.086	**0.839**	0.748
Q19	0.213	0.109	**0.811**	0.093	0.204	0.764
Q20	0.162	0.161	**0.815**	0.071	0.121	0.736
Q21	0.217	0.102	**0.813**	0.087	0.099	0.736
Q22	0.364	0.180	**0.740**	0.106	0.074	0.729

Note. Rotation method: Varimax with Kaiser normalization. Component represents extracted common factor. Only factor loadings with absolute values greater than 0.50 are presented.

### 4.3 CFA

CFA was conducted on the independent validation sample to test a first-order correlated five-factor measurement model derived from EFA. Each of the 22 scored items was specified to load only on its designated first-order factor, and the five latent factors were allowed to correlate. The attention-check item was excluded. A second-order model was not specified because the primary validation objective was to test the separability and diagnostic specificity of the five theoretically related dimensions, particularly Time Monitoring and Time Regulation. Model fit was evaluated using the chi-square/degrees-of-freedom ratio (*χ*^2^/df), root mean square error of approximation (RMSEA), comparative fit index (CFI), incremental fit index (IFI), and normed fit index (NFI). The first-order correlated model showed satisfactory fit: *χ*^2^ = 371.656, df = 199, *p* < 0.001, *χ*^2^/df = 1.8676, RMSEA = 0.037, CFI = 0.976, IFI = 0.976, and NFI = 0.949. The *χ*^2^/df ratio, incremental fit indices, and RMSEA collectively supported model fit (Kline, 2023). Results are presented in Table 4.

**Table 4:** Model fitting.

Index	Judgment criteria	Statistical value	Fitting situation
CMIN	–	371.656	–
DF	–	199	–
CMIN/DF	<3	1.868	Yes
RMSEA	<0.08	0.037	Yes
GFI	>0.90	0.950	Yes
IFI	>0.90	0.976	Yes
CFI	>0.90	0.976	Yes
RFI	>0.90	0.941	Yes
NFI	>0.90	0.949	Yes
PNFI	>0.50	0.818	Yes

The confirmatory model included all 22 scored items retained after EFA. No item was removed at the CFA stage to obtain the reported fit. Standardized factor loadings and latent-factor correlations were examined together with composite reliability and average variance extracted. Because no second-order factor was tested, the present CFA directly supports interpretation of the five correlated subscale scores; evidence for interpreting a single total score as a reflective higher-order latent construct remains to be established.

Composite reliability (CR) was used to assess the internal consistency of the indicators within each latent factor, and average variance extracted (AVE) was used to assess convergent validity. CR values of at least 0.70 and AVE values of at least 0.50 were interpreted as adequate (Fornell and Larcker, 1981; Hair et al., 2019).

As shown in Table 5, standardized factor loadings ranged from 0.653 to 0.864. All CR values exceeded 0.70 and all AVE values exceeded 0.50, supporting internal consistency and convergent validity.

**Table 5:** Convergent validity.

Path			Estimate	S.E.	C.R.	P	Std. estimate	SMC	CR	AVE
Q1	←	Time planning	1.000				0.735	0.540	0.816	0.527
Q2	←		1.069	0.060	17.909	***	0.793	0.629		
Q3	←		0.913	0.058	15.627	***	0.675	0.455		
Q4	←		1.044	0.065	16.085	***	0.696	0.485		
Q5	←	Time regulation ability	1.000				0.723	0.523	0.882	0.556
Q6	←		1.099	0.057	19.355	***	0.796	0.633		
Q7	←		0.874	0.054	16.074	***	0.661	0.437		
Q8	←		0.924	0.050	18.337	***	0.753	0.568		
Q9	←		0.942	0.056	16.795	***	0.690	0.476		
Q10	←		1.480	0.073	20.307	***	0.837	0.701		
Q11	←	Time self-efficacy	1.000				0.653	0.426	0.873	0.581
Q12	←		1.167	0.072	16.233	***	0.743	0.552		
Q13	←		1.358	0.075	18.158	***	0.864	0.747		
Q14	←		1.232	0.070	17.478	***	0.817	0.668		
Q15	←		1.056	0.067	15.759	***	0.717	0.514		
Q16	←	Sense of time value	1.000				0.808	0.654	0.801	0.574
Q17	←		0.992	0.054	18.208	***	0.768	0.590		
Q18	←		0.832	0.050	16.677	***	0.691	0.478		
Q19	←	Perception of time monitoring	1.000				0.794	0.630	0.867	0.619
Q20	←		1.129	0.054	21.013	***	0.792	0.628		
Q21	←		1.133	0.053	21.465	***	0.808	0.653		
Q22	←		0.771	0.039	19.814	***	0.753	0.567		

*p < 0.05, **p < 0.01, ***p < 0.001.

**Figure 3:**
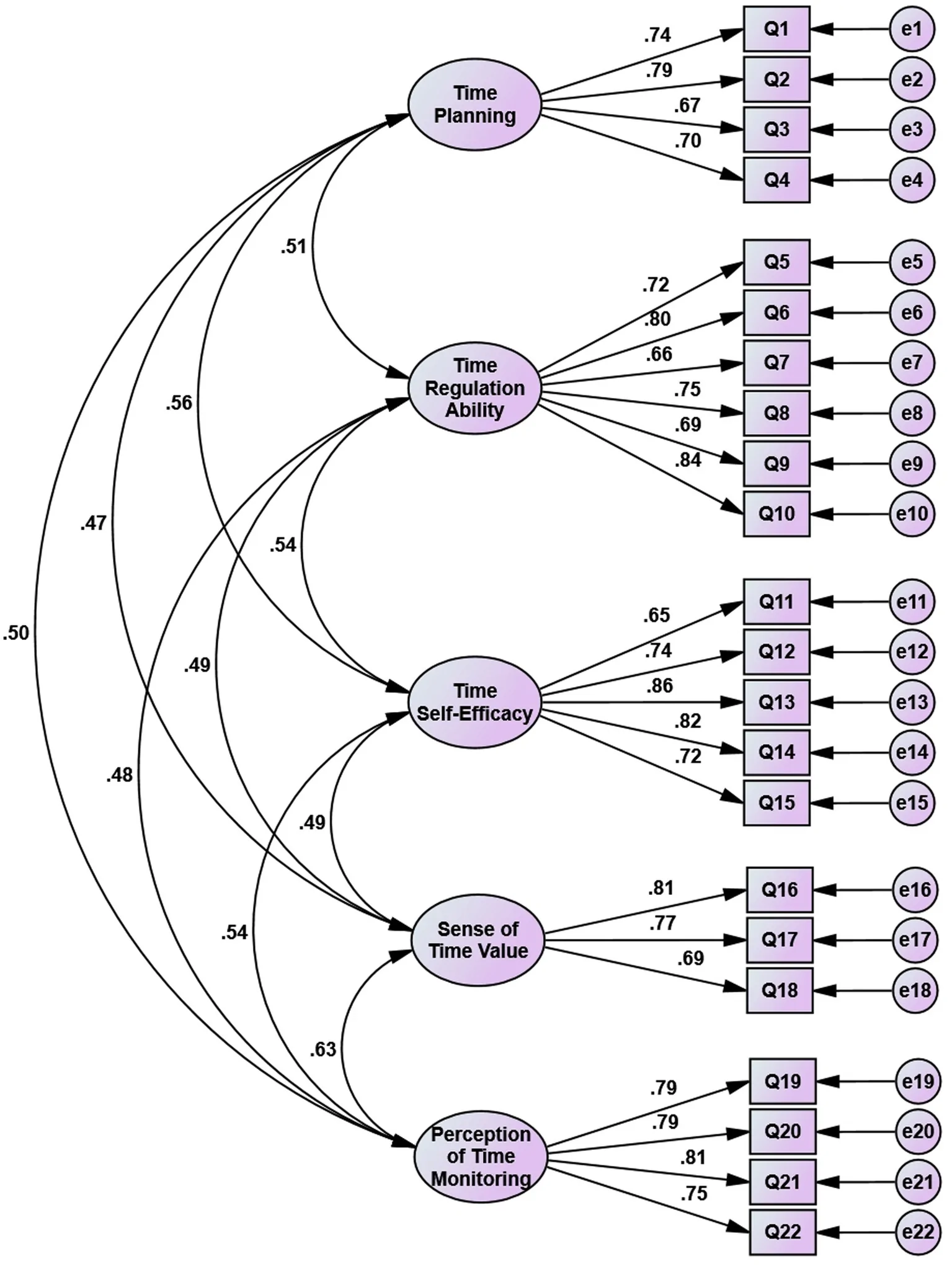
Five-factor confirmatory factor analysis model. Source: Generated by the author.

Discriminant validity was evaluated using the Fornell–Larcker criterion (Fornell and Larcker, 1981). As reported in Table 6, the square root of the average variance extracted (√AVE) for each factor exceeded its maximum absolute correlation with the other factors. Of particular relevance to the distinction between Time Monitoring and Time Regulation, the two sets of items formed separate factors in the EFA: the reported loadings ranged from 0.740 to 0.815 for Time Monitoring and from 0.664 to 0.790 for Time Regulation. The five-factor CFA also showed satisfactory global fit (*χ*^2^/df = 1.8676, RMSEA = 0.037, CFI = 0.976, IFI = 0.976, NFI = 0.949). Taken together, the content separation, independent factor loadings, and factor-level discriminant-validity results provide initial empirical support for treating monitoring and regulation as related but nonredundant constructs. These findings support the EFA-derived structure within the confirmatory framework (Figure 3).

**Table 6:** Discriminant validity.

Factors	Time planning	Time regulation ability	Time self-efficacy	Sense of time value	Perception of time monitoring
Time planning	**0.726**				
Time regulation ability	0.508***	**0.746**			
Time self-efficacy	0.559***	0.536***	**0.763**		
Sense of time value	0.468***	0.487***	0.488***	**0.757**	
Perception of time monitoring	0.497***	0.480***	0.544***	0.628***	**0.787**

Note. Bold values on the diagonal represent the square root of AVE. ***p < 0.001.

### 4.4 Reliability assessments

Cronbach’s alpha and McDonald’s omega were calculated for the total scale and each subscale. As shown in Table 7, alpha and omega were 0.813 for Factor I, 0.879 for Factor II, 0.866 for Factor III, 0.810 for Factor IV, and 0.864 for Factor V. The coefficients for the total scale were 0.95. These results indicate good internal consistency for the total scale and its five subscales.

**Table 7:** Cronbach’s alpha (*α*) and McDonald’s omega (*ω*) internal consistency coefficients.

Factors	Number of items	*α*	McDonald *ω*
*FactorsI	4	0.813	0.813
*FactorsII	6	0.879	0.879
*FactorsIII	5	0.866	0.866
*FactorsIV	3	0.810	0.810
*FactorsV	4	0.864	0.864
*SAS Total	22	0.95	0.95

## 5 Discussion and conclusion

### 5.1 Analysis of research outcomes

This research identified a five-factor framework comprising 22 scored items for the CSTMC, with initial evidence of internal consistency, convergent validity, factor-level discriminant validity, and satisfactory fit of a first-order correlated measurement model. The pilot administration also included one unscored attention-check item, which was used only for response-quality screening and is not part of the final 22-item scale. The CSTMC therefore offers a multidimensional profile of time-value beliefs, planning, monitoring, regulation, and efficacy in digitally mediated college life.

The CSTMC directly addresses contemporary time-management demands in higher education by measuring the regulatory functions required in highly autonomous, fragmented, and interruption-prone learning environments rather than digital exposure itself. Time Planning assesses students’ capacity to impose structure on flexible schedules; Time Monitoring assesses their awareness of how fragmented activities consume time; Time Regulation assesses their capacity to manage time-relevant digital interference and respond adaptively to disruptions; and Sense of Time Value and Time Efficacy capture the motivational resources needed to sustain these processes. In this way, the instrument translates the contemporary time-management challenges identified in the Introduction into separable and potentially actionable competence profiles.

The five dimensions perform complementary functions. Sense of Time Value represents beliefs about the importance of time for goal attainment and personal growth. Time Planning refers to the anticipatory allocation and prioritization of time and the translation of goals into schedules. Time Monitoring captures awareness of actual time use and task progress, including observation, recording, appraisal, and discrepancy detection without presupposing corrective action. Time Efficacy reflects confidence in managing time and attaining intended goals. Among these dimensions, Time Regulation warrants particular attention because some of its behavioral indicators may overlap with adjacent constructs such as self-control, self-discipline, and resistance to procrastination.

Time Regulation refers to the context-specific regulation of behavior and strategy in relation to temporal goals, schedules, deadlines, or time-allocation plans. It includes initiating and maintaining plan-consistent action, managing time-relevant distractions and delays, and adapting behavior when implementation deviates from the intended schedule. This dimension may share some behavioral manifestations with general self-control or self-discipline, but it is theoretically narrower because the relevant regulatory behavior is explicitly anchored to temporal goals or time-management plans. For example, the item “Once I make a plan, I take immediate action and do not let other things become excuses for procrastination” was classified as Time Regulation because it describes the initiation and maintenance of behavior in accordance with an established temporal plan. Nevertheless, its emphasis on resisting procrastination and inhibiting competing excuses may also capture a modest component of general self-control. Therefore, the boundary between Time Regulation and trait self-control requires direct empirical testing using established self-control and procrastination measures.

A central issue in interpreting the five-factor structure is why Time Monitoring and Time Regulation should remain separate despite their close temporal coupling. Consistent with the Nelson–Narens monitoring–control framework, monitoring represents diagnostic awareness of time use and task progress, whereas regulation uses temporal goals and feedback to initiate, maintain, restore, or modify the course of action (Nelson and Narens, 1990). Experimental research on self-paced study similarly operationalizes monitoring as metacognitive judgments and control as the subsequent allocation of study time, demonstrating that these processes can be measured separately while remaining functionally connected (Nelson and Leonesio, 1988). Their relationship can also be bidirectional: monitoring may guide control, while effort generated through control may provide new cues for subsequent monitoring (Koriat et al., 2006). Their continuity in real-world behavior is therefore expected, but continuity does not imply construct identity. A student may accurately detect inefficient time use yet fail to act on that information, whereas another student may make frequent adjustments based on inaccurate monitoring. The two subscales are intended to identify these different points of difficulty.

Taken together, the five dimensions form a cyclical system in which planning establishes a temporal standard, monitoring generates feedback about actual progress, and regulation governs the initiation, maintenance, restoration, or modification of action in relation to temporal goals and feedback. Accumulated experiences of success or failure may subsequently shape students’ time-value beliefs, efficacy judgments, and future planning. The importance of the five-factor solution therefore lies not only in the 68.847% of variance explained but also in its capacity to distinguish process-specific points of difficulty that would be obscured within a single broad disposition score.

The five-factor structure was evaluated using a first-order correlated confirmatory factor analysis model rather than a higher-order factor model. This specification was selected because the scale was designed to distinguish functionally different intervention targets rather than assume in advance that their shared covariance was generated by a single reflective latent factor. The fit indices (*χ*^2^/df = 1.8676, RMSEA = 0.037, CFI = 0.976, IFI = 0.976, and NFI = 0.949) supported the correlated five-factor model, and subscale reliability coefficients ranged from 0.810 to 0.879. However, these findings do not by themselves establish a second-order construct. Until a second-order or bifactor model is compared with the correlated first-order model, interpretation of the five-dimensional profile should be prioritized over strong claims based on a single total score.

The importance of the empirical pattern lies in its diagnostic implications rather than in a repetition of the fit statistics. The separate EFA factors, satisfactory correlated-factor CFA, and Fornell–Larcker results provide preliminary evidence that awareness of temporal discrepancies and action based on those discrepancies are empirically distinguishable within the proposed measurement model. This pattern is consistent with metacognitive accounts in which monitoring produces diagnostic information, whereas control governs the initiation, maintenance, or modification of behavior (Nelson and Narens, 1990; Koriat et al., 2006). Compared with the ATMD, the CSTMC therefore opens the “black box” between cognition and action by distinguishing recognition of inefficient time use from the capacity to respond adaptively. Nevertheless, because the evidence is based on cross-sectional self-report data and an alternative model combining Time Monitoring and Time Regulation was not tested, this distinction remains provisional and requires further validation.

The present study did not include an established time-management instrument or external academic criterion. Consequently, the reported AVE and Fornell–Larcker results provide evidence regarding the internal measurement structure, but they do not constitute criterion-related validity. Future validation should correlate CSTMC scores with the ATMD and related measures to examine convergent and discriminant patterns and should test associations with external outcomes such as grade point average, academic engagement, academic procrastination, assignment completion, and objectively logged time use. Longitudinal analyses are also needed to determine the predictive and incremental validity of the CSTMC beyond established measures of self-control and self-regulated learning.

### 5.2 Theoretical and practical implications

This study contributes to the literature by conceptualizing time-management competence as a dynamic, cyclical system comprising motivational, strategic, metacognitive, and behavioral functions. The five dimensions should not be interpreted as a rigid one-to-one translation of Zimmerman’s three phases; rather, they are differentiated functions distributed across and connected by the cycle. In the forethought phase, Sense of Time Value and Sense of Time Efficacy support goal engagement, while Time Planning translates goals into temporal arrangements. During performance, Time Monitoring tracks actual time use and task progress, and Time Regulation implements feedback-based changes. During self-reflection, outcomes and detected discrepancies update efficacy, value appraisals, and subsequent planning (Zimmerman, 2000, 2002). The Nelson-Narens monitoring-control framework provides a micro-level mechanism within this broader cycle (Nelson and Narens, 1990). Executive-function theory adds a complementary, but not equivalent, perspective: working memory may support the maintenance of plans and priorities, inhibitory control may support resistance to distractions, and cognitive flexibility may support strategy switching and replanning when circumstances change (Diamond, 2013). These correspondences enhance interdisciplinary interpretation, but they remain theoretical because the study did not measure executive functions or neural activity directly.

Prior research has often treated time management as a broad disposition or a relatively stable set of practices. The CSTMC extends prior student time-management assessment work (Huang and Zhang, 2001) by separating motivational beliefs from planning, monitoring, and feedback-based regulation. Cognitive load theory further suggests that excessive task demands can impair learning when students lack effective monitoring and regulation (Sweller, 1988). The CSTMC may therefore help identify whether a student’s difficulty is concentrated in planning, awareness of actual time use, adaptive adjustment, or confidence, although the diagnostic and predictive value of these profiles requires further validation.

The CSTMC may support low-stakes educational screening, advising, and intervention planning, but the present study did not establish diagnostic or criterion-referenced cutoffs. Accordingly, fixed score ranges should not yet be used to determine eligibility, impose academic sanctions, or make mental-health diagnoses. Until representative norms and criterion-linked thresholds are developed, institutions should interpret subscale profiles descriptively, compare students with appropriate local reference groups, and corroborate low scores with interviews, time-use records, academic indicators, and student consent. Freshman screening and academic early-warning use should therefore be framed as supportive case finding rather than definitive classification.

Dimension-specific profiles can nevertheless guide provisional intervention matching. Low Sense of Time Value may indicate a need for values clarification, future-goal linkage, and reflection on the personal consequences of time use. Low Time Planning may be addressed through goal decomposition, prioritization matrices, realistic scheduling, and implementation intentions. Low Time Monitoring may be addressed through time diaries, digital-use feedback, progress checkpoints, and planned comparisons between intended and actual time allocation. Low Time Regulation may call for distraction-management plans, interruption recovery routines, flexible replanning, pacing strategies, and practice in reallocating time after deviations. Low Time Efficacy may be addressed through graded mastery experiences, feedback on small successes, peer modeling, and achievable short-cycle goals. These recommendations are hypotheses for intervention design rather than validated treatment prescriptions; future studies should test whether matching interventions to subscale profiles improves outcomes.

### 5.3 Limitations and future directions

Several limitations should be considered. First, although 250 questionnaires were submitted at the pilot stage, 41 were excluded through response-quality screening, leaving 209 responses and an 83.6% usable-response rate. Specifically, 19 respondents failed the instructed-response attention-check item, 11 completed the questionnaire in less than one third of the median completion time, and 11 displayed long-string responding or near-zero response variability. These exclusions improved data quality, and the retained EFA sample met the planned item-to-sample requirement; nevertheless, excluding 16.4% of the submitted questionnaires may introduce selection bias because low-attention or low-engagement respondents may differ systematically from retained participants. The EFA sample was also smaller than the CFA sample, which may limit the stability of the exploratory solution.

Second, the EFA and CFA samples were independent but demographically similar in gender composition, grade distribution, and institution type. This similarity reduces the heterogeneity of the cross-sample test and may make replication less demanding than validation in a substantively different population. Future studies should cross-validate the structure across different geographical regions, majors, institution types, educational levels, and cultural contexts, and should test measurement invariance across these groups.

Third, content refinement relied on a single-round structured review by four experts rather than a multi-round Delphi process. The structured review improved item relevance, clarity, and dimensional correspondence, but the relatively small panel, absence of iterative consensus assessment, and lack of quantitative content-validity indices limit the strength of the content-validity evidence. Future studies should use larger and more diverse expert panels, item-level and scale-level content-validity indices, cognitive interviews with students, and a documented audit trail of expert recommendations and item decisions.

Fourth, the study did not test criterion-related validity. Future studies should examine convergence with the ATMD and other established time-management or self-regulation measures, discrimination from general self-control and personality measures, concurrent associations with academic engagement and procrastination, and predictive relations with GPA, assignment completion, retention, and objective time-use indicators. This is particularly important for determining whether Time Regulation reflects time-specific adaptive control rather than general willpower.

Fifth, only a first-order correlated five-factor CFA was tested. A second-order CFA or bifactor model should be compared with the present model to determine whether the common variance among the five dimensions supports a defensible overall time-management-competence score. Until such evidence is available, the subscale profile should be the primary interpretive unit.

Sixth, although the scale included four reverse-scored items, most scored items were positively worded. The limited use of reverse wording may have reduced respondent confusion, but it may not have fully controlled acquiescence or socially desirable responding. Moreover, reverse-worded items can themselves introduce wording-related method effects. Future research should therefore examine the balance between positively and negatively worded items and test whether item wording affects the factor structure, reliability, or response patterns. In addition, scenario-specific items concerning procrastination, distraction management, and digital-media use may be interpreted differently by students with different digital habits, learning environments, or accessibility needs. Cognitive interviewing, differential item functioning, and measurement-invariance analyses are needed to evaluate this contextual sensitivity.

Finally, the monitoring-regulation distinction and the proposed cyclical sequence were evaluated only with cross-sectional self-report data. Future studies should compare the five-factor model with a model that merges monitoring and regulation, report heterotrait-monotrait ratios, and examine differential external criteria. Experience-sampling, digital time-use logs, and observed replanning behavior could test monitoring accuracy and regulation more directly, while longitudinal and intervention designs could determine whether monitoring-generated discrepancy information predicts later regulatory action and whether profile-matched interventions improve academic outcomes.

## Data Availability

The original contributions presented in the study are included in the article/Supplementary material, further inquiries can be directed to the corresponding author/s.
