# Supplementary material for: Development and validation of the college students’ time management competence scale
Source: Front Psychol. 2026 Sep 3;17:1885044. doi: 10.3389/fpsyg.2026.1885044 (PMC13584862; doi:10.3389/fpsyg.2026.1885044)
Supplement: Supplementary file 1 [file Supplementary_file_1.docx]

Supplementary Material

**Table of Contents**

Supplementary A. TABLE1 Demographic Characteristics of Participants	2

Supplementary B. TABLE2 Results of Exploratory Factor Analysis (EFA) for the CSTMC	3

Supplementary C. TABLE3 Rotated Component Matrix	4

Supplementary D. TABLE4 Model fitting	5

Supplementary E. TABLE5 Convergent validity	6

Supplementary F. TABLE6 Discriminant validity	7

Supplementary G. TABLE7 Cronbach’s alpha (α) and McDonald’s omega (ω) internal

consistency coefficients.	8

Supplementary H. FIGURE1 Theory-driven correspondence between the SRL cycle and the five CSTMC dimensions.	9

Supplementary I. FIGURE2 Scree plot output	10

Supplementary J. FIGURE3 Five-factor confirmatory factor analysis model	11

Supplementary K. Original Questionnaire	12

Supplementary L.Table S1. Item-level expert-review decisions for the eight candidate items deleted before pilot testing	17

**Supplementary A.**

TABLE1 Demographic Characteristics of Participants

| **Items** | **Categories** | **EFA Sample (N=209)** |  | **CFA Sample (N=652)** |  |
| --- | --- | --- | --- | --- | --- |
|  |  | n | % | n | % |
| Gender | Male | 117 | 55.87 | 351 | 53.83 |
|  | Female | 92 | 44.13 | 301 | 46.17 |
| Grade | Freshman | 52 | 24.85 | 168 | 25.77 |
|  | Sophomore | 52 | 24.88 | 165 | 25.31 |
|  | Junior | 52 | 24.88 | 162 | 24.85 |
|  | Senior+ | 53 | 25.36 | 157 | 24.08 |
| Institution Type | Undergraduate | 101 | 48.32 | 320 | 49.08 |
|  | Vocational | 108 | 51.68 | 332 | 50.92 |

**Supplementary B.**

TABLE2 Results of Exploratory Factor Analysis (EFA) for the CSTMC （N=209）

| Total Variance Explained | | | | | | | | | |
| --- | --- | --- | --- | --- | --- | --- | --- | --- | --- |
| **Component** | **Initial Eigenvalues** | | | **Extraction Sums of Squared Loadings** | | | **Rotation Sums of Squared Loadings** | | |
|  | **Total** | **% of Variance** | **Cumulative %** | **Total** | **% of Variance** | **Cumulative %** | **Total** | **% of Variance** | **Cumulative %** |
| 1 | 7.695 | 34.980 | 34.980 | 7.695 | 34.980 | 34.980 | 3.860 | 17.543 | 17.543 |
| 2 | 2.415 | 10.979 | 45.958 | 2.415 | 10.979 | 45.958 | 3.283 | 14.921 | 32.464 |
| 3 | 1.902 | 8.645 | 54.603 | 1.902 | 8.645 | 54.603 | 2.956 | 13.435 | 45.899 |
| 4 | 1.600 | 7.274 | 61.877 | 1.600 | 7.274 | 61.877 | 2.652 | 12.055 | 57.954 |
| 5 | 1.533 | 6.969 | 68.847 | 1.533 | 6.969 | 68.847 | 2.396 | 10.893 | 68.847 |
| 6 | 0.702 | 3.189 | 72.035 |  |  |  |  |  |  |
| 7 | 0.640 | 2.908 | 74.943 |  |  |  |  |  |  |
| 8 | 0.606 | 2.757 | 77.700 |  |  |  |  |  |  |
| 9 | 0.539 | 2.449 | 80.149 |  |  |  |  |  |  |
| 10 | 0.512 | 2.327 | 82.476 |  |  |  |  |  |  |
| 11 | 0.461 | 2.094 | 84.570 |  |  |  |  |  |  |
| 12 | 0.432 | 1.962 | 86.531 |  |  |  |  |  |  |
| 13 | 0.416 | 1.889 | 88.420 |  |  |  |  |  |  |
| 14 | 0.365 | 1.661 | 90.081 |  |  |  |  |  |  |
| 15 | 0.349 | 1.588 | 91.669 |  |  |  |  |  |  |
| 16 | 0.335 | 1.525 | 93.194 |  |  |  |  |  |  |
| 17 | 0.304 | 1.382 | 94.576 |  |  |  |  |  |  |
| 18 | 0.286 | 1.302 | 95.878 |  |  |  |  |  |  |
| 19 | 0.283 | 1.286 | 97.164 |  |  |  |  |  |  |
| 20 | 0.230 | 1.047 | 98.211 |  |  |  |  |  |  |
| 21 | 0.208 | 0.948 | 99.159 |  |  |  |  |  |  |
| 22 | 0.185 | 0.841 | 100.000 |  |  |  |  |  |  |

**Supplementary C.**

| TABLE3 Rotated Component Matrix | | | | | | | |
| --- | --- | --- | --- | --- | --- | --- | --- |
|  | **Component** | | | | | **Extraction** | |
|  | **1** | **2** | **3** | **4** | **5** |  |  |
| Q1 | 0.117 | 0.015 | 0.169 | **0.810** | 0.134 | 0.717 | |
| Q2 | 0.031 | 0.180 | 0.107 | **0.760** | 0.245 | 0.683 | |
| Q3 | 0.198 | 0.301 | 0.086 | **0.695** | -0.054 | 0.622 | |
| Q4 | 0.130 | 0.081 | -0.023 | **0.807** | 0.007 | 0.676 | |
| Q5 | **0.664** | 0.234 | 0.269 | 0.057 | 0.192 | 0.607 | |
| Q6 | **0.790** | 0.192 | 0.199 | 0.146 | 0.090 | 0.731 | |
| Q7 | **0.751** | 0.138 | 0.078 | 0.173 | 0.197 | 0.657 | |
| Q8 | **0.728** | 0.108 | 0.263 | 0.054 | 0.241 | 0.672 | |
| Q9 | **0.737** | 0.017 | 0.163 | 0.005 | 0.084 | 0.578 | |
| Q10 | **0.777** | 0.140 | 0.149 | 0.210 | 0.077 | 0.696 | |
| Q11 | 0.148 | **0.732** | 0.164 | 0.210 | 0.145 | 0.650 | |
| Q12 | 0.190 | **0.678** | 0.116 | 0.208 | 0.021 | 0.554 | |
| Q13 | 0.129 | **0.819** | 0.128 | 0.153 | 0.209 | 0.771 | |
| Q14 | 0.070 | **0.822** | 0.141 | 0.078 | 0.158 | 0.732 | |
| Q15 | 0.115 | **0.739** | 0.028 | -0.028 | -0.140 | 0.581 | |
| Q16 | 0.266 | 0.085 | 0.155 | 0.071 | **0.818** | 0.776 | |
| Q17 | 0.219 | 0.183 | 0.159 | 0.137 | **0.779** | 0.732 | |
| Q18 | 0.145 | 0.010 | 0.122 | 0.086 | **0.839** | 0.748 | |
| Q19 | 0.213 | 0.109 | **0.811** | 0.093 | 0.204 | 0.764 | |
| Q20 | 0.162 | 0.161 | **0.815** | 0.071 | 0.121 | 0.736 | |
| Q21 | 0.217 | 0.102 | **0.813** | 0.087 | 0.099 | 0.736 | |
| Q22 | 0.364 | 0.180 | **0.740** | 0.106 | 0.074 | 0.729 | |
|  | | | | | | |  |

**Supplementary D.**

| TABLE4 Model fitting | | | |
| --- | --- | --- | --- |
| **index** | **Judgment criteria** | **Statistical value** | **Fitting situation** |
| CMIN | - | 371.656 | - |
| DF | - | 199 | - |
| CMIN/DF | <3 | 1.868 | Yes |
| RMSEA | <0.08 | 0.037 | Yes |
| GFI | >0.90 | 0.950 | Yes |
| IFI | >0.90 | 0.976 | Yes |
| CFI | >0.90 | 0.976 | Yes |
| RFI | >0.90 | 0.941 | Yes |
| NFI | >0.90 | 0.949 | Yes |
| PNFI | >0.50 | 0.818 | Yes |

**Supplementary E.**

| TABLE5 Convergent validity | | | | | | | | | | |
| --- | --- | --- | --- | --- | --- | --- | --- | --- | --- | --- |
| **Path** | | | **Estimate** | **S.E.** | **C.R.** | **P** | **Std.Estimate** | **SMC** | **CR** | **AVE** |
| Q1 | <--- | Time Planning | 1.000 |  |  |  | 0.735 | 0.540 | 0.816 | 0.527 |
| Q2 | <--- |  | 1.069 | 0.060 | 17.909 | *** | 0.793 | 0.629 |  |  |
| Q3 | <--- |  | 0.913 | 0.058 | 15.627 | *** | 0.675 | 0.455 |  |  |
| Q4 | <--- |  | 1.044 | 0.065 | 16.085 | *** | 0.696 | 0.485 |  |  |
| Q5 | <--- | Time Regulation Ability | 1.000 |  |  |  | 0.723 | 0.523 | 0.882 | 0.556 |
| Q6 | <--- |  | 1.099 | 0.057 | 19.355 | *** | 0.796 | 0.633 |  |  |
| Q7 | <--- |  | 0.874 | 0.054 | 16.074 | *** | 0.661 | 0.437 |  |  |
| Q8 | <--- |  | 0.924 | 0.050 | 18.337 | *** | 0.753 | 0.568 |  |  |
| Q9 | <--- |  | 0.942 | 0.056 | 16.795 | *** | 0.690 | 0.476 |  |  |
| Q10 | <--- |  | 1.480 | 0.073 | 20.307 | *** | 0.837 | 0.701 |  |  |
| Q11 | <--- | Time Self-Efficacy | 1.000 |  |  |  | 0.653 | 0.426 | 0.873 | 0.581 |
| Q12 | <--- |  | 1.167 | 0.072 | 16.233 | *** | 0.743 | 0.552 |  |  |
| Q13 | <--- |  | 1.358 | 0.075 | 18.158 | *** | 0.864 | 0.747 |  |  |
| Q14 | <--- |  | 1.232 | 0.070 | 17.478 | *** | 0.817 | 0.668 |  |  |
| Q15 | <--- |  | 1.056 | 0.067 | 15.759 | *** | 0.717 | 0.514 |  |  |
| Q16 | <--- | Sense of Time Value | 1.000 |  |  |  | 0.808 | 0.654 | 0.801 | 0.574 |
| Q17 | <--- |  | 0.992 | 0.054 | 18.208 | *** | 0.768 | 0.590 |  |  |
| Q18 | <--- |  | 0.832 | 0.050 | 16.677 | *** | 0.691 | 0.478 |  |  |
| Q19 | <--- | Perception of Time Monitoring | 1.000 |  |  |  | 0.794 | 0.630 | 0.867 | 0.619 |
| Q20 | <--- |  | 1.129 | 0.054 | 21.013 | *** | 0.792 | 0.628 |  |  |
| Q21 | <--- |  | 1.133 | 0.053 | 21.465 | *** | 0.808 | 0.653 |  |  |
| Q22 | <--- |  | 0.771 | 0.039 | 19.814 | *** | 0.753 | 0.567 |  |  |

* p<0.05 ** p<0.01 *** p<0.001

**Supplementary F.**

| TABLE6 Discriminant validity | | | | | |
| --- | --- | --- | --- | --- | --- |
| **Factors** | **Time Planning** | **Time Regulation Ability** | **Time Self-Efficacy** | **Sense of Time Value** | **Perception of Time Monitoring** |
| **Time Planning** | **0.726** |  |  |  |  |
| **Time Regulation Ability** | 0.508*** | **0.746** |  |  |  |
| **Time Self-Efficacy** | 0.559*** | 0.536*** | **0.763** |  |  |
| **Sense of Time Value** | 0.468*** | 0.487*** | 0.488*** | **0.757** |  |
| **Perception of Time Monitoring** | 0.497*** | 0.480*** | 0.544*** | 0.628*** | **0.787** |

Note:The bold black numbers on the diagonal represent the square root of AVE

**Supplementary G.**

TABLE7 Cronbach’s alpha (α) and McDonald’s omega (ω) internal

consistency coefficients.

| **Factors** | **Number of items** | **α** | **McDonald ω** |
| --- | --- | --- | --- |
| *FactorsⅠ | 4 | 0.813 | 0.813 |
| *FactorsⅡ | 6 | 0.879 | 0.879 |
| *FactorsⅢ | 5 | 0.866 | 0.866 |
| *FactorsⅣ | 3 | 0.810 | 0.810 |
| *FactorsⅤ | 4 | 0.864 | 0.864 |
| *SAS Total | 22 | 0.95 | 0.95 |

**Supplementary H.**

FIGURE1 Theory-driven correspondence between the SRL cycle and the five CSTMC dimensions. Sense of Time Value and Time Efficacy support goal engagement; Time Planning organizes temporal resources; Time Monitoring generates diagnostic feedback during performance; Time Regulation implements feedback-based adjustments; and reflective appraisal updates subsequent value, efficacy, and planning. The correspondence is functional rather than a strict one-to-one mapping.

**Supplementary I.**

# FIGURE2 Scree plot output

**Supplementary J.**

FIGURE3 Five-factor confirmatory factor analysis model

# Supplementary K.Original Questionnaire

College Students' Time Management Competence Scale

Instructions

Dear Student,

Hello! To understand the current status of time management competence among full-time college students, we have designed this survey. Your participation is crucial for our analysis. There are no right or wrong answers; please answer truthfully based on your actual situation. This survey will take approximately 5–10 minutes. All responses will be kept strictly confidential in accordance with the Statistics Law and used solely for academic research. Thank you for your cooperation!

**Part I: Basic Information**

1. Gender

Male

Female

2. Grade

Freshman

Sophomore

Junior

Senior+

3. Institution Type

Undergraduate

Vocational

**Part II: Self-Assessment of Time Management Competence**

(Please select the option that best reflects your actual situation. Try to avoid choosing "Moderately Agree" unless necessary.)

I. Time Planning

1. I usually break down long-term goals into actionable monthly or weekly plans.

Strongly Disagree

Somewhat Disagree

Moderately Agree

Somewhat Agree

Strongly Agree

2. I set clear goals for each important stage of my college life.

Strongly Disagree

Somewhat Disagree

Moderately Agree

Somewhat Agree

Strongly Agree

3. I have a clear idea and plan for when to study and when to relax each day.

Strongly Disagree

Somewhat Disagree

Moderately Agree

Somewhat Agree

Strongly Agree

4. I often have no set deadline for completing my tasks. (Reverse-coded)

Strongly Disagree

Somewhat Disagree

Moderately Agree

Somewhat Agree

Strongly Agree

II. Time Regulation Ability

5. Once I make a plan, I take immediate action and do not let other things become excuses for procrastination.

Strongly Disagree

Somewhat Disagree

Moderately Agree

Somewhat Agree

Strongly Agree

6. I can avoid distractions and make good use of my study time.

Strongly Disagree

Somewhat Disagree

Moderately Agree

Somewhat Agree

Strongly Agree

7. I am able to decline non-urgent matters (e.g., impromptu social events, entertainment) to protect my core study time.

Strongly Disagree

Somewhat Disagree

Moderately Agree

Somewhat Agree

Strongly Agree

8. I always schedule my most important tasks during the hours when I am most productive.

Strongly Disagree

Somewhat Disagree

Moderately Agree

Somewhat Agree

Strongly Agree

9. I often put off assigned homework until the last minute. (Reverse-coded)

Strongly Disagree

Somewhat Disagree

Moderately Agree

Somewhat Agree

Strongly Agree

10. If I notice certain activities (e.g., scrolling through short videos, gaming) are taking up too much time, I actively adjust and reduce the time spent on them.

Strongly Disagree

Somewhat Disagree

Moderately Agree

Somewhat Agree

Strongly Agree

III. Time Self-Efficacy

11. I feel confident about the goals I have set for myself.

Strongly Disagree

Somewhat Disagree

Moderately Agree

Somewhat Agree

Strongly Agree

12. I believe my allocation of time between studying and extracurricular activities is reasonable.

Strongly Disagree

Somewhat Disagree

Moderately Agree

Somewhat Agree

Strongly Agree

13. I feel that I am in control of how I spend most of my time.

Strongly Disagree

Somewhat Disagree

Moderately Agree

Somewhat Agree

Strongly Agree

14. The goals I set are often difficult to achieve. (Reverse-coded)

Strongly Disagree

Somewhat Disagree

Moderately Agree

Somewhat Agree

Strongly Agree

15. Please select “Moderately Agree” for this question to indicate that you are answering carefully. (Attention check)

Strongly Disagree

Somewhat Disagree

Moderately Agree

Somewhat Agree

Strongly Agree

16. I often feel pressed for time, unable to pursue desired activities due to other tasks. (Reverse-coded)

Strongly Disagree

Somewhat Disagree

Moderately Agree

Somewhat Agree

Strongly Agree

IV. Sense of Time Value

17. Making good use of time is highly significant to me.

Strongly Disagree

Somewhat Disagree

Moderately Agree

Somewhat Agree

Strongly Agree

18. To me, time is as precious as life itself.

Strongly Disagree

Somewhat Disagree

Moderately Agree

Somewhat Agree

Strongly Agree

19. I deeply regret the time I have wasted.

Strongly Disagree

Somewhat Disagree

Moderately Agree

Somewhat Agree

Strongly Agree

V. Perception of Time Monitoring

20. I believe time can be managed effectively.

Strongly Disagree

Somewhat Disagree

Moderately Agree

Somewhat Agree

Strongly Agree

21. I regularly review and reflect on how I use my time.

Strongly Disagree

Somewhat Disagree

Moderately Agree

Somewhat Agree

Strongly Agree

22. To improve my time efficiency, I actively seek knowledge on effective time management.

Strongly Disagree

Somewhat Disagree

Moderately Agree

Somewhat Agree

Strongly Agree

23. I routinely evaluate my plans based on whether I have achieved my goals.

Strongly Disagree

Somewhat Disagree

Moderately Agree

Somewhat Agree

Strongly Agree

Thank you for your participation!

**Supplementary L.**

Table S1. Item-level expert-review decisions for the eight candidate items deleted before pilot testing

| **Initial item (English translation)** | **Proposed dimension** | **Decision** | **Rationale for deletion** |
| --- | --- | --- | --- |
| On weekdays, I spend most of my time on entertainment. | Time Regulation | Deleted | Reverse-worded but overly broad and strongly influenced by course load, leisure norms, and daily circumstances. It assesses the amount of entertainment more than the ability to regulate time and may invite socially desirable responses. |
| I often delay completing assignments until the deadline. | Time Regulation | Deleted | Primarily reflects academic procrastination, an adjacent outcome construct, rather than time-management regulation itself. Retaining it could artificially inflate associations with procrastination measures. |
| I am always unable to complete my studies efficiently because of external interference. | Time Regulation | Deleted | Uses absolute wording ("always") and combines susceptibility to distraction with academic efficiency. The item may capture environmental constraints or general self-control rather than time-specific regulatory competence. |
| To improve the efficiency of my time use, I actively learn how to use time effectively. | Time Monitoring | Deleted | Reflects knowledge-seeking or willingness to learn time-management techniques rather than actual monitoring of time use. Its behavioral content does not directly indicate observation, recording, appraisal, or discrepancy detection. |
| I often make plans but hardly ever put them into practice. | Time Regulation | Deleted | Simultaneously assesses plan formation and implementation failure, producing ambiguous dimensional correspondence and a high risk of cross-loading between planning and regulation. |
| I believe plans can never keep pace with change, so I do not make time plans. | Time Planning | Deleted | Combines a general belief about unpredictability with a planning behavior and uses categorical wording. The response may reflect situational uncertainty or attitude toward planning rather than planning competence. |
| I believe my time does not belong to me. | Sense of Time Value | Deleted | Conceptually ambiguous and culturally or contextually sensitive. It may reflect perceived autonomy, external control, family or institutional demands, rather than the perceived value of time. |
| I often feel that I do not have enough time, but I cannot explain where the time has gone. | Time Monitoring | Deleted | Combines subjective time scarcity with poor awareness of time allocation. Because it measures both an emotional appraisal and monitoring deficiency, it is double-barrelled and overlaps with stress or workload perceptions. |

Note. The proposed dimensions refer to the initial theoretical assignment before expert review; all eight items were removed before pilot testing and were not included in item analysis, EFA, CFA, or reliability estimation.
