# Supplementary material for: Development and validation of the college students’ time management competence scale
Source: Front Psychol. 2026 Sep 3;17:1885044. doi: 10.3389/fpsyg.2026.1885044 (PMC13584862; doi:10.3389/fpsyg.2026.1885044)
Supplement: Supplementary file 2 [file Supplementary_file_2.docx]

# Supplementary K.Original Questionnaire

College Students' Time Management Competence Scale

Instructions

Dear Student,

Hello! To understand the current status of time management competence among full-time college students, we have designed this survey. Your participation is crucial for our analysis. There are no right or wrong answers; please answer truthfully based on your actual situation. This survey will take approximately 5–10 minutes. All responses will be kept strictly confidential in accordance with the Statistics Law and used solely for academic research. Thank you for your cooperation!

**Part I: Basic Information**

1. Gender

Male

Female

2. Grade

Freshman

Sophomore

Junior

Senior+

3. Institution Type

Undergraduate

Vocational

**Part II: Self-Assessment of Time Management Competence**

(Please select the option that best reflects your actual situation. Try to avoid choosing "Moderately Agree" unless necessary.)

I. Time Planning

1. I usually break down long-term goals into actionable monthly or weekly plans.

Strongly Disagree

Somewhat Disagree

Moderately Agree

Somewhat Agree

Strongly Agree

2. I set clear goals for each important stage of my college life.

Strongly Disagree

Somewhat Disagree

Moderately Agree

Somewhat Agree

Strongly Agree

3. I have a clear idea and plan for when to study and when to relax each day.

Strongly Disagree

Somewhat Disagree

Moderately Agree

Somewhat Agree

Strongly Agree

4. I often have no set deadline for completing my tasks. (Reverse-coded)

Strongly Disagree

Somewhat Disagree

Moderately Agree

Somewhat Agree

Strongly Agree

II. Time Regulation Ability

5. Once I make a plan, I take immediate action and do not let other things become excuses for procrastination.

Strongly Disagree

Somewhat Disagree

Moderately Agree

Somewhat Agree

Strongly Agree

6. I can avoid distractions and make good use of my study time.

Strongly Disagree

Somewhat Disagree

Moderately Agree

Somewhat Agree

Strongly Agree

7. I am able to decline non-urgent matters (e.g., impromptu social events, entertainment) to protect my core study time.

Strongly Disagree

Somewhat Disagree

Moderately Agree

Somewhat Agree

Strongly Agree

8. I always schedule my most important tasks during the hours when I am most productive.

Strongly Disagree

Somewhat Disagree

Moderately Agree

Somewhat Agree

Strongly Agree

9. I often put off assigned homework until the last minute. (Reverse-coded)

Strongly Disagree

Somewhat Disagree

Moderately Agree

Somewhat Agree

Strongly Agree

10. If I notice certain activities (e.g., scrolling through short videos, gaming) are taking up too much time, I actively adjust and reduce the time spent on them.

Strongly Disagree

Somewhat Disagree

Moderately Agree

Somewhat Agree

Strongly Agree

III. Time Self-Efficacy

11. I feel confident about the goals I have set for myself.

Strongly Disagree

Somewhat Disagree

Moderately Agree

Somewhat Agree

Strongly Agree

12. I believe my allocation of time between studying and extracurricular activities is reasonable.

Strongly Disagree

Somewhat Disagree

Moderately Agree

Somewhat Agree

Strongly Agree

13. I feel that I am in control of how I spend most of my time.

Strongly Disagree

Somewhat Disagree

Moderately Agree

Somewhat Agree

Strongly Agree

14. The goals I set are often difficult to achieve. (Reverse-coded)

Strongly Disagree

Somewhat Disagree

Moderately Agree

Somewhat Agree

Strongly Agree

15. Please select “Moderately Agree” for this question to indicate that you are answering carefully. (Attention check)

Strongly Disagree

Somewhat Disagree

Moderately Agree

Somewhat Agree

Strongly Agree

16. I often feel pressed for time, unable to pursue desired activities due to other tasks. (Reverse-coded)

Strongly Disagree

Somewhat Disagree

Moderately Agree

Somewhat Agree

Strongly Agree

IV. Sense of Time Value

17. Making good use of time is highly significant to me.

Strongly Disagree

Somewhat Disagree

Moderately Agree

Somewhat Agree

Strongly Agree

18. To me, time is as precious as life itself.

Strongly Disagree

Somewhat Disagree

Moderately Agree

Somewhat Agree

Strongly Agree

19. I deeply regret the time I have wasted.

Strongly Disagree

Somewhat Disagree

Moderately Agree

Somewhat Agree

Strongly Agree

V. Perception of Time Monitoring

20. I believe time can be managed effectively.

Strongly Disagree

Somewhat Disagree

Moderately Agree

Somewhat Agree

Strongly Agree

21. I regularly review and reflect on how I use my time.

Strongly Disagree

Somewhat Disagree

Moderately Agree

Somewhat Agree

Strongly Agree

22. To improve my time efficiency, I actively seek knowledge on effective time management.

Strongly Disagree

Somewhat Disagree

Moderately Agree

Somewhat Agree

Strongly Agree

23. I routinely evaluate my plans based on whether I have achieved my goals.

Strongly Disagree

Somewhat Disagree

Moderately Agree

Somewhat Agree

Strongly Agree

Thank you for your participation!
